# Effect of Buteyko breathing technique on clinical and functional parameters in adult patients with asthma: a randomized, controlled study

**DOI:** 10.1186/s40001-023-01634-1

**Published:** 2024-01-11

**Authors:** Katrin Vagedes, Silja Kuderer, Rainer Ehmann, Matthias Kohl, Johannes Wildhaber, Rudolf A. Jörres, Jan Vagedes

**Affiliations:** 1 ARCIM Institute (Academic Research in Complementary and Integrative Medicine), Filderstadt, Germany; 2 Asthma Center, Outpatient Pulmonology, Stuttgart, Germany; 3 Institute of Precision Medicine, University Furtwangen, Villingen-Schwenningen, Germany; 4 Division of Pediatrics, University and HFR, Fribourg, Switzerland; 5 Institute and Outpatient Clinic for Occupational, Social and Environmental Medicine, LMU Hospital, LMU Munich, Comprehensive Pneumology Center Munich (CPC-M), Member of the German Center for Lung Research (DZL), Munich, Germany; 6 Department of Neonatology, University Hospital Tübingen, Tübingen, Germany; 7 Department of Pediatrics, Filderklinik, Filderstadt, Germany

**Keywords:** Asthma, Self-management, Symptoms, Capnovolumetry, Respiratory medication, Buteyko breathing technique

## Abstract

**Background:**

The established therapy of asthma might be supported by additional non-pharmaceutical measures, such as the Buteyko breathing technique (BBT); however, the available data are mixed. To clarify the effects of BBT in patients with asthma, we investigated whether it led to clinical improvements with correlation to functional parameters.

**Methods:**

Using a randomized, controlled design, we studied two groups (*n* = 30 each) of patients with asthma under either BBT or usual therapy (UT) w/o BBT over a period of 3 months. The primary outcome comprised the voluntary control pause (CP) after 3 months, secondary outcomes an additional breathhold parameter, forced expiratory volume in 1 s (FEV_1_), capnovolumetry, exhaled nitric oxide (FeNO), Asthma Control Questionnaire (ACQ) and Nijmegen Questionnaire (NQ), and the use of medication (β_2_-agonists; inhaled corticosteroids, ICS).

**Results:**

CP showed significant time-by-group interaction [*F*(1,58.09) = 28.70, *p* < 0.001] as well as main effects for study group [*F*(1,58.27) = 5.91, *p* = 0.018] and time [*F*(1,58.36) = 17.67, *p* < 0.001]. ACQ and NQ scores were significantly (*p* < 0.05 each) improved with BBT. This was associated with reductions in the use of β_2_-agonists and ICS (*p* < 0.05 each) by about 20% each. None of these effects occurred in the UT group. While FEV_1_ and the slopes of the capnovolumetric expiratory phases 2 and 3 did not significantly change, the capnovolumetric threshold volume at tidal breathing increased (*p* < 0.05) with BBT by about 10 mL or 10%, compared to baseline, suggesting a larger volume of the central airways. No significant changes were seen for FeNO.

**Conclusions:**

BBT was clinically effective, as indicated by the fact that the improvement in symptom scores and the small increase in bronchial volume occurred despite the significant reduction of respiratory pharmacotherapy. As the self-controlled Buteyko breathing therapy was well-accepted by the participants, it could be considered as supporting tool in asthma therapy being worth of wider attention in clinical practice.

*Trial registration* Retrospectively registered on 10 March 2017 at ClinicalTrials.gov (NCT03098849).

## Background

Despite the widespread prevalence of asthma, which entails intensive efforts to improve its treatment, many patients experience poor control of their health condition [1, 2]. Pharmacotherapy, the treatment of choice, can be expensive, and side effects, especially of long-term corticosteroid use, are well-known [3–5]. Thus, it is a reasonable strategy to achieve best asthma control with the lowest possible medication use. This motivates the inclusion of non-pharmacological measures such as asthma education, rehabilitation and breathing training into the portfolio of treatment options. Self-management procedures, for example, comprise yoga and Tai Chi, as well as the Buteyko breathing technique (BBT) that has been reported to improve quality of life [6–8] and lung function [9–13], and to reduce asthma symptoms [7–9, 11, 12, 14, 15] and medication use [6, 7, 14, 16–19] in children and adult patients with asthma.

Buteyko hypothesized that people may be affected by the consequences of hidden chronic hyperventilation due to dysfunctional breathing, such as mouth breathing and predominant thoracic breathing. This would lead to depletion of carbon dioxide (CO_2_) and result in metabolic imbalance potentially aggravating disorders, such as asthma. The extent of hypocapnia can be estimated via a voluntary control pause (CP), i.e., the time in seconds during which a person can hold the breath with relative ease after normal expiration. Buteyko assumed that CP duration correlates with the level of alveolar and arterial carbon dioxide (PaCO_2_). Lung-healthy individuals with a relatively high value of PaCO_2_ would be capable of a longer CP compared to individuals with lower PaCO_2_ as sign of hidden chronic hyperventilation and asthma. However, research in this area is sparse and has yielded heterogeneous results [20, 21], and additional data elucidating both clinical and mechanistic aspects in parallel might be useful.

To address this question, we investigated whether the introduction of BBT in patients with asthma led to clinical improvements in symptoms and medication use that correlate with airway function. BBT was practiced at home by the patients without continuous supervision to mimic its potential practical use. Due to this, we determined adherence, i.e., whether BBT had actually been performed, via the assessment of breathhold times at the study visits. A randomized control group design with follow-up was chosen as the most powerful and adequate approach for achieving these goals. In addition, healthy control subjects were included to obtain a reference for the BBT parameters assessed.

## Methods

### Study design

We conducted a randomized controlled trial in a parallel-group design with a 1:1 allocation ratio. Participants in the Buteyko Group (BG) underwent Buteyko training as described below in addition to usual treatment, whereas the participants in the other group stayed with their usual treatment alone (UT). Data were collected at the Filderklinik (Filderstadt, Germany) and the ARCIM (**A**cademic **R**esearch in **C**omplementary and **I**ntegrative **M**edicine) Institute from January to May 2017.

### Study population

Participants were recruited through local advertisements, leaflets displayed in medical practices, and referrals from physicians. The criteria for inclusion were written informed consent; age 18 years or older; controlled, partly controlled, or uncontrolled asthma (according to NVL Asthma (German national asthma care guideline) [22, 23]); pharmacotherapy of treatment step 1 or higher [22, 23]; attending physician informed; fluency in the German language. Exclusion criteria were participation in another study; history of myocardial infarction, chronic ischemic heart disease, oncological disease, manifest mental illness. For the healthy control group (HC), acute or chronic respiratory disease was an additional exclusion criterion.

### Intervention

#### Usual treatment (UT)

Participants in this group maintained routine care, mainly consisting of standard asthma medication prescribed by the attending physicians.

#### Buteyko group (BG)

In addition to usual care as described above, these participants attended BBT training, which comprised an intensive group course on site, a booster session 1 week later, and a 3-month practice period at home. The course was given by two certified Buteyko trainers on 5 consecutive days. Each session lasted 90 min and consisted of theoretical information about normal vs. abnormal, dysfunctional breathing, e.g., breathing pattern disorders and especially hyperventilation, as well as practical training. The exercises were described in detail in a training plan given to each participant at the first session. Most of the exercises were performed in a sitting position. The participants learned to become aware of their own breathing pattern. CP was performed at the beginning and end of each exercise session and between some of the exercises. The BBT exercises comprised deliberate hypoventilation (exercises of reduced breathing) and breath-holding maneuvers (extended and maximum pauses) to regain breath control and retain CO_2_. To demonstrate that low-volume nasal breathing is also possible during physical activity, reduced breathing was also performed while walking vigorously on the spot.

In the booster session 1 week later, the trainers observed all participants during the CP and the exercises and made corrections if necessary. The participants could report about first experiences and ask questions. For the 3-month home practice, the participants were asked to practice twice per day for 20 min and to keep records of their daily adherence to the training as well as the duration (in seconds) achieved for the CP and the maximum pause (MP).

### Outcomes

Data for all outcome measures were collected by four study staff members at the examinations prior to the start of the intervention (baseline, BL) and at the 3-month follow-up (3-Mo FUP). For reasons of feasibility, measurements in both groups (BG and UT) and at both timepoints were taken at different times of day. Participants of the HC group were assessed only once, following the same protocol as in the patients with asthma.

#### Primary outcome measure

Change in CP at 3-Mo FUP. To assess CP, participants were instructed to breathe normally through the nose and, after a normal, gentle exhalation, to hold the nose closed with the thumb and forefinger and to keep the mouth closed and hold their breath until they felt air hunger. The breathing pause should only be extended long enough to allow normal nasal breathing immediately afterwards. Thereby, the length of the CP (in seconds) was recorded [20].

#### Secondary outcome measures

These comprised respiratory physiology measures, responses to questionnaires, and data on the use of asthma medication. We recorded the Buteyko maximum pause (MP), an extended breathhold performed for as long as possible until the person feels moderate discomfort [24]. Forced expiratory volume in 1 s (FEV_1_) was determined by spirometry in accordance with recommendations [25]. Capnovolumetry was performed to obtain the slopes of expiratory phases 2 (s2) and 3 (s3), their ratio s3/s2, and the threshold deadspace (VD_thre_) [26, 27]. Spirometry and capnovolumetry were performed with the device *SpiroScout*® (LF8 software, Ganshorn Medizin Electronic GmbH, Niederlauer, Germany). We used the area hyperbolic sine (areasinus hyperbolicus, asinh) as variance stabilizing and normalizing transformation in the statistical analysis of s2, s3 and s3/s2 to account for very low and zero values [28, 29]. The fractional concentration of exhaled nitric oxide was determined with the NIOX Vero device (NIOX VERO®, Circassia AG, Bad Homburg, Germany). Asthma symptoms and signs of hyperventilation were assessed by two validated questionnaires: the Asthma Control Questionnaire (ACQ) [30] and the Nijmegen Questionnaire (NQ) [31, 32]. The daily use of asthma medication was recorded by the patients. To achieve comparability, beta-2 agonist and ICS usages were converted to equivalent doses of salbutamol [6] and beclomethasone dipropionate (BDP) [2], respectively.

### Sample size

According to an a-priori power analysis based on a repeated measures ANOVA design with a power of *β* = 0.80 and a significance level of *α* = 0.05, 50 participants were needed for an effect size of *d* = 0.20. Allowing for a dropout rate of 20%, we enrolled 60 patients with asthma and an additional 30 healthy controls.

### Randomization and blinding

Patients with asthma were randomized to either the Buteyko Group (BG) or to usual treatment alone (UT). After assigning all subjects to blocks with stratification by age (number of strata = 3) and degree of asthma control (number of strata = 3), the subjects within each block were randomly assigned to the group conditions (BG or UT). To achieve equal group sizes, two opaque envelopes were prepared per block, which contained the group allocation (BG or UT). After both had been drawn, new envelopes were offered in pairs. Envelopes were offered by a research assistant not involved in the study. No further blinding was performed. To obtain values for comparison and reference, we additionally recruited age- and sex-matched healthy control individuals (HC) who were invited to a one-time measurement appointment.

### Statistical analysis

Data analysis was performed in R (version 4.2.3) [33] running in RStudio (version 2023.03.0) [34]. Missing values were treated with single imputation based on predictive mean matching (R package: mice [35]), since the proportion of missing values was small (respiration data: ≈2%, questionnaire data: ≈4%, medication data: ≈2%). We generated 40 imputed data sets and averaged them to get single imputation values. The significance level was set at *α* = 0.05 (two-tailed).

The baseline characteristics were presented as mean values and standard deviation (SD) for numerical variables along with 95% confidence intervals (95% CI) for the differences between groups. Categorical variables were expressed as absolute and relative frequency.

The primary outcome measure (change in CP at 3-Mo FUP) was analyzed via a linear mixed-effects model (R package: lme4 [36]), with subjects as a random effect and study group (BG, UT), time (BL, 3-Mo FUP) and an interaction term between study group and time as fixed effects, together with FEV_1_ as covariate. Post-hoc comparisons were performed with the R package lmerTest [37]. Holm correction was used to adjust for multiple testing within these analyses, and adjusted Cohen’s d effect sizes (*d*) were calculated (R package effsize [38]).

Secondary outcome measures not derived from the primary analysis are reported descriptively. Mean between-group differences, adjusted mean differences (to account for potential BL differences) and within-group differences as well as 95% confidence intervals and Cohen’s *d* effect sizes (*d*) were assessed for all outcome measures. All analyses referred to intention-to-treat. If appropriate, data were checked to assess whether they conformed to a normal distribution. Model assumptions were checked by residual analyses.

## Results

### Study population

**Fig. 1:**
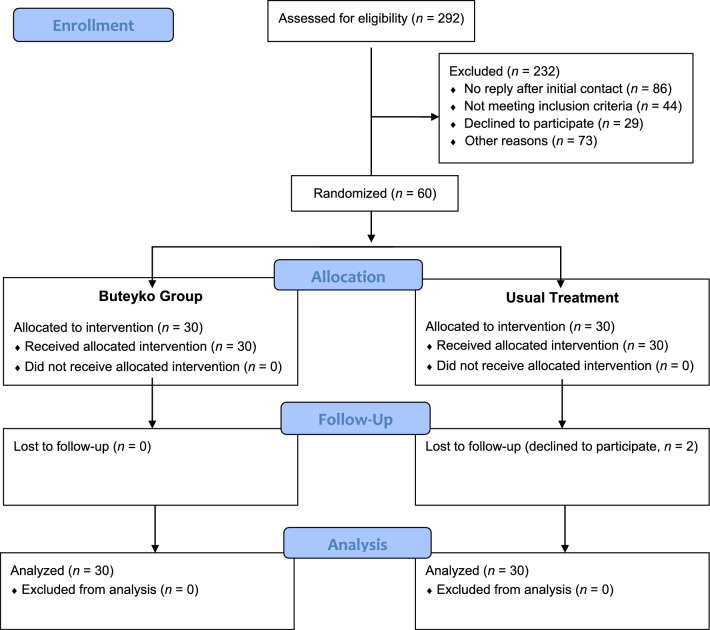
CONSORT flow diagram of the study

Initially, 292 outpatients were contacted as potential participants, of whom 60 were enrolled in the study. 232 were excluded, because they did not respond after initial contact (*n* = 86), did not meet the inclusion criteria (*n* = 44), declined to participate (*n* = 29), or for other reasons, mainly of a time or organizational nature, e.g., scheduling problems in attending the on-site Buteyko training course or excessive travel distances (*n* = 73). The 60 participants were randomly assigned to BG (*n* = 30) or UT (*n* = 30). Two of the UT participants were lost to follow-up and dropped out of the study (one for lack of time; one developed sinusitis and otitis media, was hospitalized and declined further participation) (Fig. 1).

All 60 randomized patients with asthma and all 30 HC subjects were included in the statistical analysis. 64.4% of participants were female; the mean age was 56.5 years (SD = 13.1, Min = 18, Max = 80), and the average BMI was 25.0 kg/m^2^ (SD = 4.6, Min = 16.6, Max = 46.7). The patients with asthma had received their diagnosis on average 17.4 years (SD = 14.6, Min = 1, Max = 65) prior to inclusion. In the BG, 10 patients had controlled, 11 partly controlled, and 9 uncontrolled asthma; two BG patients received step 1 pharmacotherapy, two were on step 2, 20 on step 3, and 6 on step 4 therapy. UT included 10 patients with controlled, 10 with partly controlled, and 10 with uncontrolled asthma; two UT patients were on step 1, 6 on step 2, 20 on step 3, and two on step 4 pharmacotherapy [22]. Baseline characteristics differed between HC and BG/UT but were similar between BG and UT groups, with the exception of MP and FEV_1_ (Table 1).

**Table 1:** Baseline characteristics of the study participants

	Parameters	HC (*n* = 30)	BG (*n* = 30)	UT (*n* = 30)	BG vs. HC *95% CI*†	BG vs. UT *95% CI*†	UT vs. HC *95% CI*†
Demographics	Age, years	56.4 ± 13.5	59.0 ± 13.9	53.9 ± 11.6	− 4.5; 9.7	− 1.5; 11.7	− 9.0; 4.0
	Sex, male *n* (%)†	10 (33)	10 (33)	12 (40)	–	–	–
	BMI, kg/m^2^	23.9 ± 3.6	25.4 ± 4.36	25.8 ± 5.67	− 0.53; 3.55	− 3.05; 2.09	− 0.44; 4.42
Breathhold	CP, s	18.30 ± 9.18	12.13 ± 5.96	13.83 ± 7.05	− 10.18; − 2.15	− 5.07; 1.67	− 8.70; − 0.23
	MP, s	30.77 ± 12.62	18.70 ± 6.17	24.00 ± 10.88	− 17.24; − 6.89	− 9.90; − 0.70	− 12.86; − 0.68
Spirometry	FEV_1_, %predicted	93 ± 15	69 ± 22	80 ± 16	− 34; − 14	− 21; − 1	− 21; − 5
Capnovolumetry	asinh(s2)	1.42 ± 0.25	1.38 ± 0.36	1.44 ± 0.33	− 0.20,0.12	− 0.24,0.12	− 0.13,0.17
	asinh(s3)	0.07 ± 0.05	0.19 ± 0.15	0.14 ± 0.12	**0.06,0.18**	− 0.02,0.12	**0.02,0.12**
	asinh(s3/s2)	0.04 ± 0.03	0.11 ± 0.09	0.07 ± 0.06	**0.04,0.11**	** > 0.00,0.08**	**0.01,0.06**
	Threshold deadspace, mL	92.3 ± 24.3	85.0 ± 28.0	93.7 ± 31.3	− 20.8; 6.3	− 24.0; 6.7	− 13.1; 15.9
Symptoms	Asthma Control Questionnaire (ACQ), score	–	2.03 ± 1.11	1.64 ± 1.13	–	− 0.18; 0.97	–
	Nijmegen Questionnaire (NQ), score	3.53 ± 5.07	19.3 ± 10.1	15.6 ± 7.4	11.6; 19.9	− 0.9; 8.3	8.8; 15.4
Medication	β_2_-Agonists (salbutamol equivalents), µg/d	–	290.5 ± 193.6	310.5 ± 267.8	–	− 141.1; 101.0	–
	ICS (BDP equivalents), µg/d	–	875.8 ± 849.0	740.8 ± 593.0	–	− 244.5; 514.4	–
Inflammation	FeNO, ppb	20.2 ± 11.8	31.7 ± 23.3	35.8 ± 33.1	2.0; 21.2	− 18.9; 10.7	2.7; 28.7

Data are mean values ± SD or †numbers and percentages. *HC*  healthy controls, *BG*  Buteyko group, *UT*  usual treatment, *BMI*  body mass index, *CP*  control pause, *MP*  maximum pause, *FEV*_*1*_  forced expiratory volume in 1 s, *s2*  slope of phase 2 in g/mol*L, *s3*  slope of phase 3 in g/mol*L, *s3/s2*  ratio of slopes of phases 3 and 2, *asinh*  Areasinus hyperbolicus, *ACQ*  Asthma Control Questionnaire, *NQ*  Nijmegen Questionnaire, *ICS*  inhaled corticosteroids, *BDP*  beclomethasone dipropionate, *FeNO*  fractional exhaled nitric oxide. Bold type indicates significant effects or differences with confidence intervals that do not contain zero

Apart from the one UT participant hospitalized for sinusitis and otitis media, no adverse events were reported.

### Primary and secondary outcome measures

#### Primary outcome measure

The primary analysis regarding CP yielded a significant time-by-group interaction [*F*(1,58.09) = 28.70, *p* < 0.001] as well as a statistically significant main effect for study group [*F*(1,58.27) = 5.91, *p* = 0.018] and time [*F*(1,58.36) = 17.67, *p* < 0.001]. With respect to post-hoc analyses, Holm adjustment revealed no significant difference between both groups at BL (estimate = − 1.63, 95% CI − 4.82, 1.56; *p*_adj_ = 0.94, *d*_adj_ = − 0.15) but a significant increase within BG (estimate = − 8.65, 95% CI − 11.21, − 6.08; *p*_adj_ < 0.001, *d*_adj_ = − 0.81) as well as a significant between-group difference at 3-Mo FUP (estimate = 8.05, 95% CI 4.89, 11.21; *p*_adj_ < 0.001, *d*_adj_ = 0.74) (data in detail: Table 2). In this analysis FEV_1_ was included as covariate due to the baseline differences between groups, while MP was not included due to its close relationship to and collinearity with CP.

**Table 2:** Post-hoc comparison of time-by-group interactions

Group-by-time combinations	Estimate	95% CI (lower)	95% CI (upper)	*p* value	Holm-adjusted *p* value	Cohen’s *d* adjusted
BG_BL_ vs. UT_BL_	− 1.63	− 4.82	1.56	0.31	0.94	− 0.15
BG_BL_ vs. BG_3-Mo FUP_	− 8.65	− 11.21	− 6.08	< 0.001	< 0.001	− 0.81
BG_BL_ vs. UT_3-Mo FUP_	− 0.59	− 3.79	2.61	0.71	0.94	0.06
UT_BL_ vs. BG_3-Mo FUP_	− 7.01	− 10.17	− 3.86	< 0.001	< 0.001	− 0.66
UT_BL_ vs. UT_3-Mo FUP_	1.04	− 1.52	3.59	0.42	0.94	0.10
BG_3-Mo FUP_ vs. UT_3-Mo FUP_	8.05	4.89	11.21	< 0.001	< 0.001	0.74

The table shows post-hoc comparisons of all possible time-by-group interaction combinations along with adjusted Cohen’s *d* and *p* values

The results regarding changes in CP corresponded to those of comparisons with the BL measurement of HC. At BL, HC had significantly higher values compared to BG (*d* = − 0.80) and UT (*d* = − 0.55). At 3-Mo FUP, BG values were no longer different from BL measurements of the HC (*d* = 0.33), whereas in the UT group values were still lower than those of the HC (*d* = − 0.73) (Table 5).

#### Secondary outcome measures

*Functional measures* At BL, MP was significantly lower in BG than in UT (*d* = − 0.60) (Table 3) but at the 3-Mo FUP, MP was higher in BG compared to UT (*d* = 1.27) (Table 3), corresponding to a significant within-increase in BG (*d* = 1.66) (Table 4). Accordingly, MP differed between HC and BG at BL, but not at the 3-Mo FUP. In contrast, UT showed significantly lower values at both BL and 3-Mo FUP, compared to HC (Table 5).

**Table 3:** Adjusted mean differences between parameters in Buteyko (BG) and usual treatment group (UT)

Parameters	Timepoint	BG (*n* = 30)	UT (*n* = 30)	Mean difference†	95% CI †	ES †	Adj. mean difference‡	95% CI ‡	ES ‡
*Functional indices*									
CP, s	BL	12.13 ± 5.96	13.83 ± 7.05	− 1.70	− 5.07; 1.67	− 0.26	**9.70**	6.09; 13.31	1.39
	3-Mo FUP	20.80 ± 5.70	12.80 ± 5.37	**8.00**	5.14; 10.86	1.44			
MP, s	BL	18.70 ± 6.17	24.00 ± 10.88	**− 5.30**	− 9.90; − 0.70	− 0.60	**17.07**	11.98; 22.16	1.73
	3-Mo FUP	33.30 ± 10.76	21.53 ± 7.43	**11.77**	6.97; 16.56	1.27			
FEV_1_, %predicted	BL	69 ± 22	80 ± 16	− 11	− 21; − 1	− 0.56	3	− 4; 9	0.22
	3-Mo FUP	73 ± 21	81 ± 19	− 8	− 19; 2	− 0.41			
asinh(s2)	BL	1.38 ± 0.36	1.44 ± 0.33	− 0.06	− 0.24,0.12	− 0.18	0.06	− 0.12,0.24	0.18
	3-Mo FUP	1.37 ± 0.30	1.37 ± 0.38	0.00	− 0.18,0.18	0			
asinh(s3)	BL	0.19 ± 0.15	0.14 ± 0.12	0.05	− 0.02,0.12	0.38	− 0.04	− 0.11,0.02	− 0.33
	3-Mo FUP	0.16 ± 0.11	0.15 ± 0.14	0.01	− 0.05,0.07	0.08			
asinh(s3/s2)	BL	0.11 ± 0.09	0.07 ± 0.06	**0.04**	> 0.00,0.08	0.52	**− 0.03**	− 0.07, < 0.00	− 0.53
	3-Mo FUP	0.09 ± 0.07	0.08 ± 0.08	0.01	− 0.03,0.04	0.08			
Threshold deadspace, mL	BL	85.0 ± 28.0	93.7 ± 31.3	− 8.7	− 24.0; 6.7	− 0.29	**11.0**	0.01; 21.9	0.52
	3-Mo FUP	95.3 ± 27.9	93.09 ± 26.9	2.3	− 11.9; 16.5	0.08			
*Symptoms*									
ACQ, score	BL	2.03 ± 1.11	1.64 ± 1.13	0.39	− 0.18; 0.97	0.35	**− 0.48**	− 0.88; − 0.09	− 0.63
	3-Mo FUP	1.47 ± 0.99	1.56 ± 1.11	− 0.09	− 0.63; 0.45	− 0.09			
NQ, score	BL	19.3 ± 10.3	15.6 ± 7.4	3.7	− 0.9; 8.3	0.42	**− 3.8**	− 7.5; − 0.02	− 0.52
	3-Mo FUP	11.0 ± 9.2	11.1 ± 8.2	− 0.05	− 4.6; 4.5	− 0.01			
*Medication*									
β_2_-Agonists (salbutamol equivalents), µg/d	BL	290.5 ± 193.6	310.5 ± 267.8	− 20.0	− 141.1; 101.0	− 0.09	− 68.6	− 144.6; 7.5	− 0.47
	3-Mo FUP	226.9 ± 201.1	315.6 ± 288.1	− 88.6	− 217.3; 40.1	− 0.36			
ICS (BDP equivalents), µg/d	BL	875.8 ± 849.0	740.8 ± 593.0	135.0	− 244.5; 514.4	0.18	**− 245.5**	− 463.1; − 27.9	− 0.58
	3-Mo FUP	714.3 ± 857.2	824.9 ± 625.3	− 110.6	− 499.1; 278.1	− 0.15			
FeNO	BL	31.7 ± 23.3	35.8 ± 33.1	− 4.1	− 18.9; 10.7	− 0.14	4.77	− 4.9; 14.5	0.26
	3-Mo FUP	37.0 ± 28.1	36.3 ± 30.4	0.7	− 14.5; 15.8	0.02			

Values refer to baseline (BL) and 3-month follow-up (3-Mo FUP). Moreover, between-group differences as a function of time are shown. Data are mean values and S*D*. † between-group differences at BL and 3-Mo FUP. ‡ Adjusted between-group differences. *ES*  Cohen’s *d*, *CI*  confidence interval, *Adj.*  adjusted, *CP*  control pause, *MP*  maximum pause, *FEV*_*1*_  forced expiratory volume in 1 s, *s2*  slope of phase 2 in g/mol*L, *s3*  slope of phase 3 in g/mol*L, *s3/s2*  ratio of slopes of phases 3 and 2, *asinh*  Areasinus hyperbolicus, *ACQ*  Asthma Control Questionnaire, *NQ*  Nijmegen Questionnaire, *ICS*  inhaled corticosteroids, *BDP*  beclomethasone dipropionate, *FeNO*  fractional exhaled nitric oxide. Bold type indicates significant effects or differences with confidence intervals that do not contain zero

**Table 4:** Within-group differences for outcome parameters in Buteyko (BG) and usual treatment group (UT)

	Parameters	BG: 3-Mo FUP vs. BL			UT: 3-Mo FUP vs. BL		
		Mean difference	95% CI	ES	Mean difference	95% CI	ES
Breathhold	CP, s	**8.67**	6.15; 11.19	1.49	− 1.03	− 3.73; 1.66	− 0.16
	MP, s	**14.60**	10.75; 18.45	1.66	− 2.47	− 5.97; 1.03	− 0.26
Spirometry	FEV_1_, %predicted	3	− 1; 8	0.16	1	− 4; 5	0.04
Capnovolumetry	asinh(s2)	− 0.01	− 0.12,0.10	− 0.03	− 0.07	− 0.22,0.08	− 0.2
	asinh(s3)	− 0.03	− 0.09,0.03	− 0.23	0.01	− 0.03,0.05	0.09
	asinh(s3/s2)	− 0.02	− 0.05,0.00	− 0.3	0.01	− 0.01,0.03	0.15
	Threshold deadspace, mL	**10.3**	3.3; 17.3	0.37	− 0.7	− 9.4; 8.1	− 0.02
Symptoms	ACQ, score	**− 0.56**	− 0.88; − 0.24	− 0.54	− 0.08	− 0.33; 0.17	− 0.07
	NQ, score	**− 8.3**	− 11.0; − 5.5	− 0.85	**− 4.5**	− 7.1; − 1.9	− 0.58
Medication	β_2_-Agonists (salbutamol equivalents), µg/d	**− 63.5**	− 112.0; − 15.1	− 0.32	5.1	− 55.67; 65.7	0.02
	ICS (BDP equivalents), µg/d	**− 161.5**	− 317.5; − 5.5	− 0.19	84.1	− 74.4; 242.5	0.14
Inflammation	FeNO, ppb	5.2	− 3.1; 13.5	0.2	0.5	− 4.9; 5.8	0.01

The table shows within-group differences for outcome parameters in Buteyko group (BG) and usual treatment group (UT) as a function of time (BL = baseline, 3-Mo FUP = 3-month follow-up). Within-group differences refer to 3-Mo FUP vs. BL. *ES*  Cohen’s *d*, *CI*  confidence interval, *CP*  control pause, *MP*  maximum pause, *FEV*_*1*_  forced expiratory volume in 1 s, *s2*  slope of phase 2 in g/mol*L, *s3*  slope of phase 3 in g/mol*L, *s3/s2*  ratio of slopes of phases 3 and 2, *asinh*  Areasinus hyperbolicus, *ACQ*  Asthma Control Questionnaire, *NQ*  Nijmegen Questionnaire, *ICS*  inhaled corticosteroids, *BDP*  beclomethasone dipropionate, *FeNO*  fractional exhaled nitric oxide. Bold type indicates significant effects or differences with confidence intervals that do not contain zero

**Table 5:** Between-group differences: healthy controls (HC) vs. Buteyko (BG) or usual treatment group (UT)

	Parameters	Time	HC vs. BG			HC vs. UT		
			Mean difference	95% CI	ES	Mean difference	95% CI	ES
Breathhold	CP, s	BL	**− 6.17**	− 10.18; − 2.15	− 0.80	**− 4.47**	− 8.70; − 0.23	− 0.55
		3-Mo FUP	2.50	− 1.47; 6.47	0.33	**− 5.50**	− 9.41; − 1.59	− 0.73
	MP, s	BL	**− 12.07**	− 17.24; − 6.89	− 1.21	**− 6.77**	− 12.86; − 0.68	− 0.57
		3-Mo FUP	2.53	− 3.53; 8.60	0.22	**− 9.23**	− 14.61; − 3.85	− 0.89
Spirometry	FEV_1_, %predicted	BL	**− 0.24**	− 0.34; − 0.14	− 1.26	**− 0.13**	− 0.21; − 0.05	− 0.83
		3-Mo FUP	**− 0.21**	− 0.30; − 0.11	− 1.11	**− 0.12**	− 0.21; − 0.04	− 0.73
Capnovolumetry	asinh(s2)	BL	− 0.04	− 0.20, 0.12	− 0.13	0.02	− 0.13, 0.17	0.07
		3-Mo FUP	− 0.05	− 0.19, 0.09	− 0.18	− 0.05	− 0.22, 0.12	− 0.16
	asinh(s3)	BL	**0.12**	0.06, 0.18	1.08	**0.07**	0.02, 0.12	0.74
		3-Mo FUP	**0.09**	0.05, 0.14	1.07	**0.08**	0.03, 0.13	0.79
	asinh(s3/s2)	BL	**0.08**	0.04, 0.11	1.16	**0.04**	0.01, 0.06	0.73
		3-Mo FUP	**0.05**	0.03, 0.08	1.03	**0.05**	0.02, 0.08	0.8
	Threshold deadspace, mL	BL	− 7.3	− 20.8; 6.3	− 0.28	1.4	− 13.1; 15.9	0.05
		3-Mo FUP	3.0	− 10.5; 16.6	0.12	0.7	− 12.5; 14.0	0.03
Symptoms	NQ, score	BL	**15.8**	11.7; 19.9	1.97	**12.1**	8.8; 15.4	1.90
		3-Mo FUP	**7.5**	3.6; 11.4	1.01	**7.6**	4.0; 11.1	1.11
Inflammation	FeNO, ppb	BL	**11.6**	2.0; 21.2	0.63	**15.7**	2.7; 28.7	0.63
		3-Mo FUP	**16.8**	5.5; 28.1	0.78	**16.1**	4.1; 28.2	0.70

The table shows between-group differences between healthy controls (HC, *n* = 30) and either Buteyko group (BG, *n* = 30) or usual treatment group (UT, *n* = 30). Differences refer to the values at baseline (BL) or 3-month follow-up (3-Mo FUP). *ES*  Cohen’s *d*, *CI*  confidence interval, *CP*  control pause, *MP*  maximum pause, *FEV1*  forced expiratory volume in 1 s, *s2*  slope of phase 2 in g/mol*L, *s3*  slope of phase 3 in g/mol*L, *s3/s2*  ratio of slopes of phases 3 and 2, *asinh* Areasinus hyperbolicus, *NQ*  Nijmegen Questionnaire, *FeNO*  fractional exhaled nitric oxide. The Asthma Control Questionnaire was omitted as it could not be sensibly asked in the HC group; the same was true for medication. Bold type indicates significant effects or differences with confidence intervals that do not contain zero

BG had slightly lower baseline values of FEV_1_ than UT (Table 3), despite randomization. Neither BG nor UT showed significant within-changes over time (Table 4). Compared with HC, BG and UT had a significantly lower FEV_1_ at both BL and 3-Mo FUP (Table 5).

**Fig. 2:**
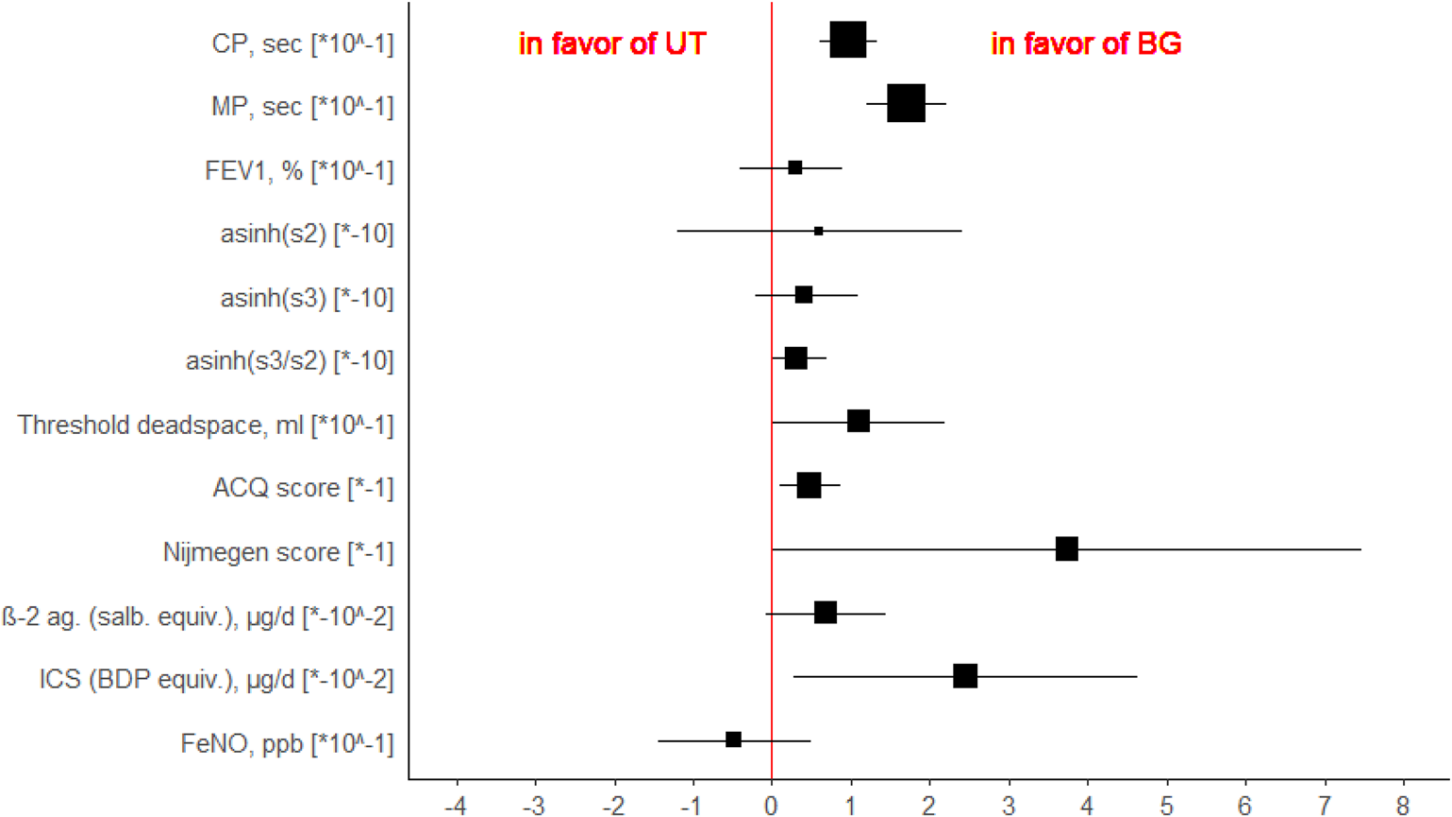
Adjusted mean differences between the BBT and the UT group as given in Table 3 together with their 95% confidence intervals. The numbers in the brackets indicate conversion factors used to render the magnitude of the outcome measures better readable without using different scales

Regarding capnovolumetry we found no significant difference for asinh(s2) and asinh(s3) but a significant adjusted mean difference for asinh(s3/s2) indicating an improvement approximating HC for the Buteyko group (Table 3, Fig. 2). HC showed significantly lower values for asinh(s3) and asinh(s3/s2), compared with BG and UT (Table 5). The values of VD_thre_ in BG and UT did not differ from those of HC, neither at BL nor at 3-Mo FUP (Table 5). In the longitudinal comparison, however, VD_thre_ increased significantly from BL to 3-Mo FUP in BG (with a small ES, Table 4) but not in UT, resulting in a significant adjusted mean difference (Table 3, Fig. 2).

With respect to FeNO, BG and UT had higher NO values than HC at BL and 3-Mo FUP (Table 5). No significant within- or between differences were found for BG and UT (Tables 3, 4).

*Questionnaires and medication* Regarding the ACQ score, BG showed a significant decrease between BL and 3-Mo FUP, with a medium effect size (Table 4). Although the differences BG vs. UT were not significant at both BL and 3-Mo FUP, we found a significant effect for the adjusted mean difference regarding the comparison of changes in the two groups (*d* = − 0.63) (Table 3, Fig. 2).

With respect to the NQ score, both BG and UT showed significant decreases over time, but with higher effect sizes for BG (Table 4), as reflected in a significant adjusted mean difference of changes between BG and UT (*d* = − 0.52) (Table 3, Fig. 2). Compared to HC, the score was elevated in BG and UT, both at BL and 3-Mo FUP, with high effect sizes (Table 5).

Regarding the use of beta-2 agonists and ICS, BG showed a reduction between BL and 3-Mo FUP, with small effect sizes. In contrast, medication intake remained almost constant for UT (Table 4). No significant differences between BG and UT were found either at BL or 3-Mo FUP, but the adjusted mean difference of the changes over time turned out to be significant for ICS intake (*d* = − 0.58) (Table 3, Fig. 2).

## Discussion

In our study of the effects of the Buteyko breathing technique (BBT) in adults with asthma, we found improvements in several outcome parameters in the Buteyko group. CP, a basic breathhold measure, and MP increased as direct consequences of BBT indicating adherence to the therapy at home. Importantly, BBT also had clinical effects, since asthma control in terms of ACQ and NQ improved, while asthma medication use was reduced. However, at the end of the study period NQ was still higher than in healthy control subjects, indicating that the asthma was—understandably—not eliminated by BBT. Spirometry did not indicate changes in airway function, in contrast to capnovolumetry at resting ventilation, as the threshold deadspace slightly increased in the BBT but not in the Usual Treatment group, suggesting an increase in bronchial volume possibly indicating a better functional status. Taken together, the results of this comprehensive study revealed small but clear clinical benefits of the Buteyko breathing technique (BBT) in adult patients with asthma, which were reflected by both clinical and functional parameters.

Buteyko suggested CO_2_ depletion from chronic hyperventilation as a factor contributing to the development or aggravation of asthma, and proposed the assessment of the corresponding hypocapnia via CP in a simple procedure. Courtney and Cohen [29] tested Buteyko’s hypothesis in 83 adults who had normal or abnormal spirometry and were categorized as either healthy or suspected of dysfunctional breathing. They found a negative correlation between CP and end-tidal CO_2_, in contrast to Buteyko’s original assumption, and no difference in end-tidal CO_2_ between individuals with abnormal vs. normal spirometry. Despite this, CP was shorter in the abnormal spirometry group and correlated with a dysfunctional breathing pattern [20]. Bowler and colleagues observed low end-tidal CO_2_ in patients with asthma and no change with BBT or a control condition (placebo breathing technique) [16]. In contrast, Abramson et al. found an increase in end-tidal CO_2_ with BBT that was greater than with asthma education [39]. Slader and colleagues compared BBT-like exercises with non-specific upper body exercises and observed no differences in end-tidal CO_2_ or changes within the groups [19]. In a study of patients with asthma divided into a medication plus BBT and a medication-only group, Hassan et al. found an improvement in CP in the BBT group but not in the medication-only group [9]. El-Nahas et al. [31] investigated the effect of BBT on acid–base balance in asthma. Patients were assigned to either a BBT group or a group with standard chest physiotherapy and assessed before and after 8 weeks of training. The outcome measures were arterial blood gas (ABG) analysis, CP, and an asthma questionnaire. The BBT group showed significant within-group improvements in ABG, CP, and questionnaire data, in contrast to the physiotherapy group. After treatment, between-group differences were also in favor of BBT [40].

The present study used different types of outcome measures to substantiate a potential effect of BBT. The first one comprised two indices of breathhold capacity directly linked to BBT exercises. The fact that these indices were changed in the Buteyko group confirms that the exercises at home were performed by the participants and that, therefore, an essential condition for a valid assessment of effects was satisfied. The second type of assessments comprised clinical measures in terms of asthma control and need for respiratory medication. These were of major importance, since they demonstrated the clinical effectiveness of the BBT. Obviously, it would be of minor relevance if BBT would change breathing parameters without clinical correlates. The third set of outcome assessments comprised physiological indices. We assessed FEV_1_ as an established measure of lung function. It mainly served for patient characterization, as we did not expect major effects of BBT on FEV_1_. However, just the absence of changes was relevant in showing that the reduction in medication use was not associated with a deterioration of conventional lung function parameters. More subtle information was expected from capnovolumetry during resting ventilation, as it might capture slight changes in the inhomogeneity of ventilation or in bronchial volume [26, 27]. Indeed, the threshold volume, which is the expired volume until the first rise in exhaled CO_2_ occurs, became larger after BBT but not after usual therapy. This might be interpreted as sign of a slight increase in bronchial volume which could be an indicator of persistent bronchodilation [41] that could not be observed in the less sensitive FEV_1_.

To assess potential effects on airway inflammation, we determined the exhaled nitric oxide in terms of FeNO. This is an easily measurable marker of Th2-type airway inflammation produced by epithelial cells as a result of IL-13-induced activation of nitric oxide synthase [42–44]. FeNO is associated with the clinical response to inhaled corticosteroids (ICS) and with airway hyperresponsiveness (AHR) [45–48]. Correspondingly, it has a significant role in the diagnosis of asthma [49], although its precise value depends on a number of variables, such as age, height, smoking status and atopic condition [46, 50–53]. In the present study we did not expect a reduction of FeNO with BBT, and in fact a slight, but not statistically significant increase was observed, while the value in the UT group remained constant. The increase was probably due to the reduction of ICS use in the BBT group and, remarkably enough, not linked to lower but to better asthma control. This result appears to underline an ICS-sparing potential of BBT without compromising asthma control. Independent of effects of BBT by itself, it might also be that the awareness associated with the daily exercises improved the patients’ ability to adjust the ICS therapy according to their needs without eliciting an impairment in asthma control. If true, this would be an interesting side-effect of the Buteyko approach.

The BBT resulted in, albeit small, clinically measurable improvements, and these reached the minimal clinically important difference of 0.5 points in the ACQ. The NQ turned out to be less informative despite showing a large mean change, due to its much larger variation. Remarkably, with BBT the use of both beta-2 agonists and inhaled corticosteroids was reduced by about 20% after 3 months compared to baseline, whereas ACQ and the use of medication were virtually unchanged in the patients receiving usual treatment. It should also be noted that none of the randomly allocated patients of the BBT group complained about the additional efforts and time required. In view of this, it seems a remarkable success that using a simple non-pharmacological intervention, the clinical state of asthma was improved and at the same time, the need for respiratory medication reduced. While this might be of minor importance regarding beta-2 agonists, it might be relevant for corticosteroids given their adverse side effects that have been demonstrated in patients with asthma [3–5, 54, 55], apart from the reduction in medication costs.

Due to possible diurnal variability in symptoms and lung function, patients with asthma should be examined at the same time of day [2]. For reasons of feasibility, assessments in both groups (BG and UT) and at both timepoints (BL and 3-Mo FUP) were carried out at different times of the day, which is a limitation of the present study. To rule out changes in measurement times as a confounding factor, we analyzed the times and found no significant differences between the groups and the timepoints. We, therefore, assume that the changing measurement times might have affected both groups and timepoints equally and did not influence our results.

Although the number of included participants satisfied the demands from the power calculation and was sufficient to observe statistically significant effects, it was too small to allow for the identification of potential subgroups of responders and non-responders to BBT, a further limitation of our study. It would also be of interest to study patients with severe asthma to reveal whether BBT elicits positive effects even in these patients who have a high demand for therapy and in whom psychological problems are not rare.

## Conclusions

The data of this randomized, controlled trial demonstrated positive clinical effects in terms of improved asthma control and lower medication use after Buteyko breathing therapy at home over a period of 3 months. These improvements were associated with improvements of indices used for the quantification of breathhold prolongation with Buteyko therapy. They were also reflected in a small increase in bronchial volume detected by capnovolumetry during normal breathing, which again occurred despite the reduction in respiratory pharmacotherapy. As the self-controlled breathing therapy was well-accepted by the participants, it seems to be a supporting tool in asthma therapy that is worth of wider attention in clinical practice.

## Data Availability

The data used to support the findings of this study are available from the corresponding author on reasonable request.

## Abbreviations

ACQ: Asthma Control Questionnaire
asinh: Areasinus hyperbolicus
BBT: Buteyko breathing technique
BDP: Beclomethasone dipropionate
BG: Buteyko group
BL: Baseline
CP: Control pause (Buteyko control pause in seconds)
FeNO: Fractional concentration of exhaled nitric oxide
FEV_1_: Forced expiratory volume in 1 s
HC: Healthy controls
ICS: Inhaled corticosteroids
MP: Maximum pause (Buteyko maximum pause in seconds)
NQ: Nijmegen Questionnaire
PaCO_2_: Alveolar CO_2_ partial pressure
s2: Slope of phase 2 in capnovolumetry
s3: Slope of phase 3 in capnovolumetry
3-Mo FUP: 3-Month follow-up
UT: Usual treatment
VD_thre_: Threshold deadspace
